# Supplementary material for: Hot spot prediction in protein-protein interactions by an ensemble system
Source: BMC Syst Biol. 2018 Dec 31;12(Suppl 9):132. doi: 10.1186/s12918-018-0665-8 (PMC6311905; doi:10.1186/s12918-018-0665-8)
Supplement: Supplementary file 2 — All relevant MATLAB code. A simple Matlab implement of our predictor. (ZIP 19,742 kb) [file 12918_2018_665_MOESM2_ESM.zip › Additional file 2/Read ASA/ASA Property/SKEMPI/2HRKA.txt]

 NetSurfP - Protein Surface Accessibility and Secondary Structure Predictions

  

|  |  |  |
| --- | --- | --- |
|  |  | NetSurfP - Protein Surface Accessibility and Secondary Structure Predictions  Technical University of Denmark |

  


---

```
# For publication of results, please cite:
# A generic method for assignment of reliability scores applied to solvent accessibility predictions.
# Bent Petersen, Thomas Nordahl Petersen, Pernille Andersen, Morten Nielsen and Claus Lundegaard
# BMC Structural Biology 2009, 9:51 doi:10.1186/1472-6807-9-51
#
# Column 1: Class assignment - B for buried or E for Exposed - Threshold: 25% exposure, but not based on RSA
# Column 2: Amino acid
# Column 3: Sequence name
# Column 4: Amino acid number
# Column 5: Relative Surface Accessibility - RSA
# Column 6: Absolute Surface Accessibility
# Column 7: Z-fit score for RSA prediction
# Column 8: Probability for Alpha-Helix
# Column 9: Probability for Beta-strand
# Column 10: Probability for Coil
E P  2HRKA                  1    0.770 109.306   0.476   0.003   0.003   0.994
E S  2HRKA                  2    0.317  37.106  -1.640   0.005   0.262   0.733
E T  2HRKA                  3    0.487  67.547   0.668   0.004   0.616   0.381
B L  2HRKA                  4    0.125  22.961   0.429   0.003   0.718   0.279
E T  2HRKA                  5    0.428  59.364   0.458   0.002   0.816   0.182
B I  2HRKA                  6    0.121  22.348   0.527   0.003   0.718   0.279
E N  2HRKA                  7    0.361  52.836   0.318   0.005   0.336   0.660
E G  2HRKA                  8    0.445  35.029  -1.118   0.018   0.047   0.935
E K  2HRKA                  9    0.737 151.642  -0.155   0.018   0.019   0.964
B A  2HRKA                 10    0.258  28.410  -0.749   0.018   0.019   0.964
E P  2HRKA                 11    0.508  72.071  -0.216   0.058   0.017   0.925
E I  2HRKA                 12    0.396  73.316  -1.153   0.181   0.016   0.803
B V  2HRKA                 13    0.120  18.505  -0.220   0.430   0.016   0.555
B A  2HRKA                 14    0.112  12.386  -1.262   0.522   0.016   0.462
B Y  2HRKA                 15    0.109  23.379   0.354   0.782   0.003   0.216
B A  2HRKA                 16    0.090   9.885  -0.220   0.923   0.002   0.076
B E  2HRKA                 17    0.060  10.517   0.027   0.970   0.001   0.030
B L  2HRKA                 18    0.064  11.682   0.507   0.975   0.003   0.022
B I  2HRKA                 19    0.046   8.492   0.790   0.975   0.003   0.022
B A  2HRKA                 20    0.034   3.747  -0.287   0.975   0.003   0.022
B A  2HRKA                 21    0.049   5.356   0.232   0.975   0.003   0.022
E R  2HRKA                 22    0.307  70.326   0.573   0.938   0.007   0.055
B I  2HRKA                 23    0.133  24.642   0.854   0.879   0.010   0.111
B V  2HRKA                 24    0.061   9.391  -0.204   0.751   0.050   0.199
E N  2HRKA                 25    0.535  78.324   0.916   0.561   0.047   0.393
E A  2HRKA                 26    0.349  38.438   0.018   0.354   0.048   0.598
E L  2HRKA                 27    0.527  96.475  -0.298   0.184   0.043   0.773
E A  2HRKA                 28    0.279  30.735   0.372   0.113   0.043   0.844
E P  2HRKA                 29    0.659  93.455  -0.288   0.053   0.043   0.903
E N  2HRKA                 30    0.626  91.573  -1.201   0.018   0.047   0.935
E S  2HRKA                 31    0.618  72.441  -0.628   0.004   0.138   0.858
B I  2HRKA                 32    0.153  28.249   0.342   0.004   0.420   0.576
E A  2HRKA                 33    0.457  50.350   1.182   0.002   0.816   0.182
B I  2HRKA                 34    0.087  16.114   0.246   0.001   0.900   0.099
E K  2HRKA                 35    0.352  72.345   0.890   0.001   0.959   0.040
B L  2HRKA                 36    0.203  37.169   0.624   0.001   0.900   0.099
E V  2HRKA                 37    0.412  63.324  -0.349   0.002   0.816   0.182
E D  2HRKA                 38    0.446  64.341  -0.291   0.022   0.552   0.426
E D  2HRKA                 39    0.520  74.946  -1.287   0.018   0.088   0.893
E K  2HRKA                 40    0.521 107.108  -0.427   0.018   0.047   0.935
E K  2HRKA                 41    0.561 115.295  -0.106   0.019   0.141   0.840
B A  2HRKA                 42    0.203  22.404  -0.726   0.018   0.088   0.893
E P  2HRKA                 43    0.657  93.157  -1.070   0.018   0.088   0.893
E A  2HRKA                 44    0.604  66.550  -1.651   0.019   0.141   0.840
E A  2HRKA                 45    0.451  49.700  -0.230   0.022   0.359   0.619
E K  2HRKA                 46    0.305  62.759   0.595   0.022   0.552   0.426
E L  2HRKA                 47    0.357  65.385   0.155   0.023   0.655   0.322
B D  2HRKA                 48    0.193  27.869  -0.581   0.022   0.552   0.426
E D  2HRKA                 49    0.490  70.595  -1.195   0.021   0.279   0.699
E A  2HRKA                 50    0.348  38.317  -1.328   0.018   0.088   0.893
B T  2HRKA                 51    0.221  30.694  -0.846   0.339   0.016   0.645
E E  2HRKA                 52    0.367  64.045  -0.941   0.802   0.014   0.185
E D  2HRKA                 53    0.276  39.743  -0.036   0.923   0.002   0.076
B V  2HRKA                 54    0.028   4.334   0.510   0.975   0.003   0.022
B F  2HRKA                 55    0.033   6.563   0.494   0.975   0.003   0.022
E N  2HRKA                 56    0.306  44.754   1.884   0.970   0.001   0.030
B K  2HRKA                 57    0.124  25.486   1.149   0.988   0.000   0.012
B I  2HRKA                 58    0.025   4.625   1.095   0.970   0.001   0.030
B T  2HRKA                 59    0.081  11.207   0.643   0.970   0.001   0.030
E S  2HRKA                 60    0.546  63.944   1.502   0.923   0.002   0.076
E K  2HRKA                 61    0.295  60.599   1.051   0.858   0.002   0.139
B F  2HRKA                 62    0.115  23.040   0.454   0.600   0.003   0.397
E A  2HRKA                 63    0.466  51.320  -0.080   0.321   0.003   0.675
E A  2HRKA                 64    0.647  71.266   0.578   0.502   0.002   0.495
E I  2HRKA                 65    0.275  50.819  -0.472   0.522   0.016   0.462
E F  2HRKA                 66    0.360  72.332  -1.019   0.257   0.016   0.727
E D  2HRKA                 67    0.546  78.707  -0.992   0.058   0.017   0.925
E N  2HRKA                 68    0.523  76.626  -0.923   0.053   0.043   0.903
E G  2HRKA                 69    0.615  48.424  -1.958   0.115   0.016   0.868
E D  2HRKA                 70    0.541  78.016  -0.035   0.181   0.016   0.803
E K  2HRKA                 71    0.470  96.761   0.882   0.858   0.002   0.139
E E  2HRKA                 72    0.524  91.455   0.400   0.923   0.002   0.076
E Q  2HRKA                 73    0.284  50.758   1.251   0.923   0.002   0.076
B V  2HRKA                 74    0.046   7.132   0.587   0.970   0.001   0.030
E A  2HRKA                 75    0.377  41.512   0.540   0.970   0.001   0.030
E K  2HRKA                 76    0.438  90.117   0.928   0.970   0.001   0.030
B W  2HRKA                 77    0.056  13.516   0.310   0.970   0.001   0.030
B V  2HRKA                 78    0.104  15.985   1.034   0.970   0.001   0.030
E N  2HRKA                 79    0.431  63.098   0.199   0.923   0.002   0.076
B L  2HRKA                 80    0.178  32.592   1.139   0.970   0.001   0.030
B A  2HRKA                 81    0.059   6.480   0.036   0.970   0.001   0.030
B Q  2HRKA                 82    0.268  47.918  -0.300   0.923   0.002   0.076
E K  2HRKA                 83    0.464  95.548  -0.656   0.858   0.002   0.139
E E  2HRKA                 84    0.369  64.412   0.065   0.600   0.003   0.397
B L  2HRKA                 85    0.178  32.628  -2.383   0.321   0.003   0.675
E V  2HRKA                 86    0.304  46.694  -0.321   0.181   0.016   0.803
E I  2HRKA                 87    0.496  91.741  -0.687   0.058   0.017   0.925
E K  2HRKA                 88    0.348  71.584  -0.068   0.058   0.017   0.925
E N  2HRKA                 89    0.680  99.581   0.001   0.058   0.017   0.925
E F  2HRKA                 90    0.270  54.209   0.144   0.782   0.003   0.216
E A  2HRKA                 91    0.646  71.156   0.896   0.858   0.002   0.139
E K  2HRKA                 92    0.473  97.317   0.756   0.858   0.002   0.139
B L  2HRKA                 93    0.045   8.313  -0.112   0.970   0.001   0.030
E S  2HRKA                 94    0.319  37.410   0.920   0.970   0.001   0.030
E Q  2HRKA                 95    0.542  96.855   1.530   0.970   0.001   0.030
B S  2HRKA                 96    0.137  16.056   0.586   0.970   0.001   0.030
B L  2HRKA                 97    0.037   6.683   0.538   0.970   0.001   0.030
E E  2HRKA                 98    0.493  86.197   2.180   0.970   0.001   0.030
E T  2HRKA                 99    0.436  60.515   1.670   0.970   0.001   0.030
B L  2HRKA                100    0.049   8.972   0.941   0.970   0.001   0.030
B D  2HRKA                101    0.212  30.506   0.589   0.923   0.002   0.076
E S  2HRKA                102    0.626  73.332   1.652   0.858   0.002   0.139
B Q  2HRKA                103    0.248  44.346   1.322   0.802   0.014   0.185
B L  2HRKA                104    0.059  10.766   0.709   0.622   0.015   0.363
E N  2HRKA                105    0.328  48.034   0.026   0.339   0.016   0.645
E L  2HRKA                106    0.499  91.367  -0.068   0.191   0.086   0.723
E R  2HRKA                107    0.303  69.318   0.328   0.118   0.150   0.732
B T  2HRKA                108    0.131  18.100  -0.272   0.066   0.296   0.638
B F  2HRKA                109    0.128  25.750   0.680   0.079   0.592   0.329
B I  2HRKA                110    0.083  15.355  -0.256   0.079   0.592   0.329
B L  2HRKA                111    0.065  11.902  -0.311   0.074   0.484   0.442
B G  2HRKA                112    0.258  20.305  -1.085   0.056   0.142   0.802
E G  2HRKA                113    0.512  40.294  -1.063   0.018   0.047   0.935
B L  2HRKA                114    0.243  44.493   0.257   0.053   0.043   0.903
E K  2HRKA                115    0.455  93.491   0.540   0.191   0.086   0.723
B Y  2HRKA                116    0.082  17.545   1.108   0.278   0.093   0.628
B S  2HRKA                117    0.081   9.470   0.204   0.386   0.097   0.517
B A  2HRKA                118    0.100  10.987   0.154   0.938   0.007   0.055
B A  2HRKA                119    0.053   5.885   0.207   0.975   0.003   0.022
B D  2HRKA                120    0.051   7.320   0.187   0.975   0.003   0.022
B V  2HRKA                121    0.034   5.180   0.710   0.975   0.003   0.022
B A  2HRKA                122    0.056   6.160   0.629   0.975   0.003   0.022
B C  2HRKA                123    0.027   3.861   0.928   0.975   0.003   0.022
B W  2HRKA                124    0.033   7.912   0.697   0.975   0.003   0.022
B G  2HRKA                125    0.060   4.738  -0.255   0.938   0.007   0.055
B A  2HRKA                126    0.108  11.935   0.084   0.938   0.007   0.055
B L  2HRKA                127    0.041   7.525   0.119   0.802   0.014   0.185
B R  2HRKA                128    0.228  52.097   0.759   0.717   0.014   0.269
E S  2HRKA                129    0.439  51.474  -0.509   0.430   0.016   0.555
B N  2HRKA                130    0.192  28.036  -0.583   0.257   0.016   0.727
E G  2HRKA                131    0.396  31.205  -0.674   0.268   0.043   0.689
E M  2HRKA                132    0.401  80.280  -0.615   0.278   0.093   0.628
B C  2HRKA                133    0.266  37.318  -1.058   0.386   0.097   0.517
B G  2HRKA                134    0.275  21.658  -0.696   0.386   0.097   0.517
E S  2HRKA                135    0.541  63.452  -0.395   0.354   0.048   0.598
E I  2HRKA                136    0.493  91.205  -0.767   0.354   0.048   0.598
E I  2HRKA                137    0.349  64.583  -0.489   0.268   0.043   0.689
E K  2HRKA                138    0.538 110.605  -0.282   0.181   0.016   0.803
E N  2HRKA                139    0.568  83.184  -0.178   0.115   0.016   0.868
E K  2HRKA                140    0.590 121.281  -1.261   0.058   0.017   0.925
E V  2HRKA                141    0.557  85.580   0.064   0.058   0.017   0.925
B D  2HRKA                142    0.160  23.070   0.700   0.058   0.017   0.925
E V  2HRKA                143    0.357  54.825   0.295   0.181   0.016   0.803
B N  2HRKA                144    0.126  18.417  -0.448   0.660   0.049   0.291
B V  2HRKA                145    0.032   4.934   0.253   0.938   0.007   0.055
B S  2HRKA                146    0.254  29.781   0.613   0.938   0.007   0.055
B R  2HRKA                147    0.266  60.868   1.314   0.970   0.001   0.030
B W  2HRKA                148    0.042  10.005   0.532   0.970   0.001   0.030
B Y  2HRKA                149    0.111  23.657   0.426   0.970   0.001   0.030
E T  2HRKA                150    0.493  68.435   1.036   0.970   0.001   0.030
B L  2HRKA                151    0.157  28.692   1.283   0.923   0.002   0.076
B L  2HRKA                152    0.046   8.459   0.431   0.858   0.002   0.139
E E  2HRKA                153    0.355  62.071   0.378   0.694   0.003   0.303
E M  2HRKA                154    0.427  85.403  -0.610   0.502   0.002   0.495
B D  2HRKA                155    0.203  29.310   1.144   0.406   0.004   0.590
E P  2HRKA                156    0.543  77.023  -0.515   0.694   0.003   0.303
E I  2HRKA                157    0.535  99.030   0.567   0.694   0.003   0.303
B F  2HRKA                158    0.059  11.881   0.034   0.923   0.002   0.076
E G  2HRKA                159    0.393  30.961   1.545   0.923   0.002   0.076
E E  2HRKA                160    0.608 106.148   1.750   0.970   0.001   0.030
B A  2HRKA                161    0.055   6.083  -0.324   0.970   0.001   0.030
B H  2HRKA                162    0.122  22.137   0.031   0.923   0.002   0.076
E D  2HRKA                163    0.678  97.729   1.137   0.858   0.002   0.139
E F  2HRKA                164    0.473  94.891   1.298   0.782   0.003   0.216
B L  2HRKA                165    0.083  15.271   0.993   0.502   0.002   0.495
E S  2HRKA                166    0.472  55.260   0.387   0.502   0.002   0.495
E K  2HRKA                167    0.546 112.374   0.179   0.600   0.003   0.397
E S  2HRKA                168    0.481  56.420   0.024   0.694   0.003   0.303
B L  2HRKA                169    0.216  39.568  -0.139   0.782   0.003   0.216
E L  2HRKA                170    0.528  96.768   0.710   0.858   0.002   0.139
E E  2HRKA                171    0.615 107.423   0.425   0.782   0.003   0.216
B L  2HRKA                172    0.168  30.761   0.168   0.694   0.003   0.303
E K  2HRKA                173    0.440  90.529   0.608   0.600   0.003   0.397
E K  2HRKA                174    0.615 126.485   0.580   0.600   0.003   0.397
E S  2HRKA                175    0.536  62.866  -0.179   0.502   0.002   0.495
E A  2HRKA                176    0.379  41.788  -1.330   0.321   0.003   0.675
E N  2HRKA                177    0.911 133.429   0.567   0.003   0.003   0.994

Explain the output.  Go back.
```

---
